# Anatomical study and early diagnosis of dome galls in *Cordia Dichotoma* using DeepSVM model

**DOI:** 10.3389/frai.2025.1558358

**Published:** 2026-01-05

**Authors:** Said Khalid Shah, Mazliham Bin Mohd Su’ud, Aurangzeb Khan, Muhammad Mansoor Alam, Muhammad Ayaz

**Affiliations:** 1 Department of Computer Science, University of Science and Technology, Bannu, Khyber Pakhtunkhwa, Pakistan; 2 Department of Computer Science, Multimedia University Cyberjaye Campus, Persiaran Multimedia, Cyberjaya, Malaysia; 3 Department of Computer Science, Riphah International University, Islamabad, Pakistan

**Keywords:** classification, *Cordia dichotoma*, DeepSVM, dome galls, fine tuning, Resnet-50, SVM, transfer learning

## Abstract

**Introduction:**

Artificial intelligence (AI), particularly deep learning (DL), offers automated solutions for early detection of plant diseases to improve crop yield. However, training accurate models on real-field data remains challenging due to over fitting and limited generalization. As observed in prior studies, traditional CNNs often struggle with real-environment variability, and transfer learning can lead to instability in training on domain-specific leaf datasets. This study focuses on detecting dome galls, a disease in *Cordia dichotoma*, by formulating a binary classification task (healthy vs. diseased leaves) using a custom dataset of 3,900 leaf images collected from real field environments.

**Methods:**

Initially, both custom CNNs and transfer learning models were trained and compared. Among them, a modified ResNet-50 architecture showed promising results but suffered from over fitting and unstable convergence. To address this, the final sigmoid activation layer was replaced with a Support Vector Machine (SVM), and L2 regularization was applied to reduce over fitting. This hybrid DeepSVM architecture stabilized training and improved model robustness. Image preprocessing and augmentation techniques were applied to increase variability and prevent over fitting.

**Results:**

The final model was evaluated on a separate test set of 400 images, and the results remained stable across repeated runs. DeepSVM achieved an accuracy of 94.50% and an F1-score of 94.47%, outperforming other well-known models like VGG-16, InceptionResNetv2, and MobileNet-V2.

**Conclusion:**

These results indicate that the proposed DeepSVM approach offers better generalization and training stability than conventional CNN classifiers, potentially aiding in automated disease monitoring for precision agriculture.

## 1 Introduction

Agriculture is a cornerstone of global food security and economic stability, yet crop productivity remains highly vulnerable to pathogen-induced diseases. However, plants are vulnerable to a range of illnesses caused by pests and pathogens, leading to an estimated $200 million in global economic losses annually. With the global population increasing by 1.6% each year, the demand for food and agricultural products continues to rise (Ashwinkumar et al., 2022). Experts plan to adopt new methods and technologies to detect and identify plant diseases at early stages to save the loss caused by plant diseases (Ullah et al., 2023). However, diseases such as dome galls in *Cordia dichotoma* remain understudied despite their economic impact, necessitating dedicated data-driven detection frameworks. After the enhancement in artificial intelligence-based techniques, like machine learning (ML) and deep learning (DL), the automated classification of image data, including plant diseases at early stages, has been a hot research area for the last decade (Bhosale et al., 2024). Various types of convolutional neural networks (CNNs) models from scratch and with transfer learning have been trained and deployed in real environments with satisfactory results (Altalak et al., 2022; Zhu et al., 2024).

Compared to previous naked-eye classifications, which were time-consuming and less satisfactory, the new automated methods produce accurate and timely results without the involvement of field experts because they can be used by non-expert users with a smartphone or drone cameras (Li et al., 2019). Early identification of dome galls in *Cordia dichotoma* leaves is essential due to their negative impact on crop yields and economic losses. Conventional detection methods lack speed and precision, necessitating a novel approach. By employing DL models customized for dome gall detection, this research offers a transformative solution to quickly identify and mitigate the spread of this specific disease. This advancement is vital for preserving crop health, ensuring sustainable agriculture, and meeting global food demands (Bhosale et al., 2023).

In the proposed study, dome galls, a disease that occurs in *Cordia dichotoma*, were addressed. The genus Cordia belongs to the family Boraginaceae of plants (Bhattacharya and Saha, 2013). It includes about 300 species of trees and shrubs, most of which are indigenous to warmer regions of the world. It is utilized in various industries, including medicine, agriculture, office supplies, musical instruments, furniture, watercraft, painting, and energy (Ferahtia, 2021; Ganjare et al., 2011; Matias et al., 2015). Dome galls are a common disease of *Cordia dichotoma* affecting the leaves in the form of multiple dome-like structures. The literature I reviewed indicates that no one has previously addressed this problem, and there is no publicly available dataset on the Internet. Therefore, images were collected from real scenarios for training and testing the DeepSVM model. Various villages in Bannu district, Khyber Pakhtunkhwa, Pakistan, were visited, and a custom data set was created. The image data was divided into two categories: dome galls and normal images. Most plant leaf disease symptoms are based on color change or wilting, but the target disease is unique in structure and has multiple raised surface areas called dome galls. It needs more concentration to classify the early stage of the disease because it is very close to healthy leaves, and it is difficult for ML and DL models to classify it easily. To increase the performance of the proposed model, training data, preprocessing and data augmentation techniques play an important role.

The proposed study used the transfer learning technique with ResNet-50 as the backbone for the plant leaf disease classification model, using pre-trained weights from the ImageNet dataset. To adapt the model for the specific task, three fully connected (FC) layers were added on top of the ResNet50. SVM was used as the final/output layer instead of the sigmoid layer to identify the leaf images as healthy or infected. The study’s key contributions include the following:

- A novel dataset on Cordia leaves is created, comprising 3,500 images taken from a real environment and labeled into two categories: healthy and diseased
- The study embraces a novel approach by fusing morpho-anatomical insight and automated computational systems to understand the root cause of the diseases better
- The leaves were subjected to careful morpho-anatomical examination using stereo and light microscopy. The disease was diagnosed as the mites-induced dome-galls on the leaf surface
- The novel DeepSVM model was trained and fine-tuned based on transfer learning to differentiate between healthy leaves and the dome gall’s early symptoms
- The performance of the DeepSVM model was evaluated with that of previous state-of-the-art (SOTA) models and two publicly available datasets to identify the generalizability of the proposed approach

The rest of the paper is arranged as follows: Section II reviews the relevant literature, while Section III outlines the methods used in our study. In Section IV, we present results and discussions. Finally, we offer some concluding thoughts and suggestions for future research in Section V

## 2 Related work

Due to ongoing developments in DL and computer vision, experts are attempting to integrate these new technologies in various fields including the agriculture industry (Zhu et al., 2025). They are used in agriculture for multiple purposes, such as plant diseases, identification of plant species, pest detection fruit ripeness, etc. CNN is a type of DL used for picture classification, image segmentation, object detection, and recognition. Most previous research work based on automatic plant disease detection and classification utilized CNN models with transfer learning. Pre-trained models (VGG-16, ResNet-50, MobileNet, EfficientNet, DenseNet, Inception, etc.) are used mostly for transfer learning because they are conducive to generalizing an image classification model if the training dataset is small. In contrast, training from scratch is only needed when there are several thousand training images, which is an arduous task (Hungilo et al., 2019).

Jakjoud et al. (2019) trained VGG-16 with various optimizers, including SGD, Adagrad, RMSprop, and Adadelta. They used a dataset of 13,692 images split in the ratio of 80% for training and 20% for testing, with 100 ×100 as the input size. They concluded that Adadelta was the most accurate, while SGD was the fastest and came in second in accuracy. Zekiwos and Bruck (2021) used a cotton leaf dataset that contained 2,400 images with 4 classes: one healthy and three diseased. Augmentation techniques were used to enhance the training process. Pre-processing techniques like image resizing and normalization, were utilized. Three convolutional layers for feature extraction and two fully connected layers for classification were used to train a customized CNN model. They trained the model with and without augmentation and claimed a 15% increase in accuracy with data augmentation. K-fold cross-validation was used. Adam and RMSprop optimizers were used, and Adam was recommended with 96.4% accuracy. Nandhini and Ashokkumar (2021) used a tomato leaf image dataset of 11,942 images of one healthy class and nine diseased classes taken from PlantVillage. In the Keras package, they created a CNN model with three convolutional layers, max pooling, two dense layers, and one output layer for classification. Ghosal and Sarkar (2020) built a CNN model for rice leaf disease classification. They used VGG-16 as the backbone of the model for transfer learning to overcome the drawback of a small training dataset. The model was trained using a small custom dataset consisting of 1,649 photos divided into four classes. Enhancing the data was utilized to lessen over-fitting and claim 92.4% accuracy. Bhujel and Shakya (2022) used a leaf picture data of rice taken from Kaggle containing 524 photos for each of the 4 categories. They used CNN model with transfer learning and EfficientB0 and EfficientNetB3 as base frameworks with new fine-tuned FC layers. They used the cyclical learning rate (CLR). They claimed 83.99 and 89.18% accuracy after 15 epochs, respectively.

A pre-trained network, DenseNet-121, was used by Chakraborty et al. (2021) to identify plant leaf ailments. For collecting training images, a well-known platform called PlanDoc was used. Multiple optimization techniques were used while fine-tuning the proposed approach, but SGD was finalized due to its stable growth while training. They claimed 92.5% accuracy after 10 epochs. Krishnamoorthy et al. (2021) trained a CNN model with transfer learning. InceptionResNetV2 was used as the backbone of the model. They used 1,000 images as a training set and 300 images for validation collected from Kaggle based on 4 classes. Image augmentation and preprocessing techniques were used to generalize the model. Dropout, batch normalization and global average pooling were used in the head of the model to reduce over fitting. They claimed 95.67% accuracy on the unseen images called test datasets. Hong et al. (2020) used transfer learning. They trained five tomato leaf disease classification models, including ResNet-50, Xception, MobileNet, ShuffleNet, and DenseNet121-Xception, with nine disease classes and a healthy class. ResNet-50 was recommended as the best model after applying image preprocessing and data augmentation. The training set consists of 13,112 photos downloaded from PlantVillage.

Kumar and Vani (2019) trained six models, Xception, LeNet, EfficientNet, VGG-16, VGG-19 and ResNet-50, with fine-tuning for tomato disease classification. The models have trained on color and grayscale segmented images separately with the SGD optimizer, and their results were compared. They claimed that VGG-16 performed well with color images, achieving 99.5% accuracy. Shijie et al. (2017) used a CNN model based on transfer learning. The VGG-16 model was used as the backbone of the model. Data augmentation techniques were used and achieved 89% accuracy. They used a tomato leaf dataset with 400 images per class and divided it into 65% for training, 25% for validation, and 10% for testing. They also tested the model with SVM as the final layer but got 1% less accuracy. They used 40 as the batch size, a 0.001 learning rate, and an SGD optimizer with 80 epochs.

Chowdhury et al. (2021) replaced the GoogleNet backbone with a new backbone made up of numerous convolutional layers, batch normalization (BN), and max pooling. They trained their custom backbone on the Plant-Village dataset and compared the results to the GoogleNet model’s output. They claimed that their model performed well compared to the original model. DeepPlantNet is a novel 28-layer deep learning model that consists of three FC layers and twenty-five convolutional layers, as presented by Ullah et al. (2023). The distinctive and successful Plant disease classification system uses Leaky RelU, BN, fire modules, and a combination of 3 × 3 and 1 × 1 filters. With average accuracy rates for eight-class and three-class classification schemes of 98.49 and 99.85%, respectively, DeepPlantNet successfully classified plant illnesses into ten categories. This novel method helps experts and farmers quickly detect and treat plant illnesses, which presents a viable way to lower agricultural losses. In a study by Sujatha et al. (2021), several pre-built ML and DL models were compared on a custom dataset of citrus leaves comprising five categories. The results showed that deep learning surpassed machine learning approaches, with random forest (RF) producing the lowest precision and VGG-Net producing the highest precision.

Syed-Ab-Rahman et al. (2022) employed transfer learning to achieve 94.37 per cent accuracy by utilizing a faster RCNN model trained on citrus leaf images accessible on Kaggle. Roy and Bhaduri (2021) used a modified version of Yolov4 with transfer learning on a dataset of apple leaves with a dataset of three classes and achieved 91.2% accuracy. They used image augmentation approach to expand the training set and prepare the model for complex backdrop images. Ullah et al. (2022) suggest using DeepPestNet, an end-to-end deep learning network, to identify and categorize crop pests. There are 11 layers in the model, including 8 convolution layers and 3 dense layers. The authors used image augmentation techniques such as rotation, flipping, blurring etc. to expand the training set and evaluate the robustness of the suggested method. Using crops dataset of Deng, they used 10 classes of pests with a success rate of 100%, and the Kaggle pest dataset did it with an accuracy of 98.92%.

Jamjoom et al. (2023) used conventional ML methods for plant leaf disease classification. For data preprocessing, discrete cosine transformation (DCT) and color space conversions were used. To segment the training images, a famous approach known as K-means clustering was used, and local binary pattern (LBP) feature grey level co-occurrence matrix (GLCM) was used for feature extraction. Radial base kernel and polynomial kernel approaches were used for feature classification, and SVM was used for disease type identification. Arshad et al. (2023) used preprocessing techniques including data augmentation and the U-Net model for region-of-interest segmentation. CNN models Vgg-19 and Inception v3 as ensemble learning were used for feature extraction, and transformers were used for the identification of potato diseases. CNN is considered to be the building block of recent computer vision applications. Bhatti et al. (2023) used a custom CNN model equipped with preprocessing and data augmentation for feature extraction and transfer learning based on Inception-V3 for disease identification. The proposed study used a hybrid training dataset taken from Plant-Village and Plant-DOC with sixty classes and claimed 99% testing accuracy.

The design of a good model for early diagnosis of plant leaf diseases requires a great knowledge of related literature to know the hardness of the area. Except for a few diseases for which datasets are available on the internet, there is a scarcity of dataset images for plant diseases. Collecting target disease leaf images from real-world environments is time-consuming and labor-intensive. Training a generalized model for such a new disease requires deep knowledge of CNN structure and parameters and their role in training a model. In the next section, all these issues are addressed systematically.

## 3 Methodology

The proposed approach comprises two main steps, i.e., anatomical and computational studies. The anatomical study foresees the root causes of possible diseases in the plant leaves. Similarly, the computational study uses fine-tuned CNN with SVM to classify the *Cordia dichotoma* leaves into healthy and diseased. The details of each methodological study are elaborated below:

### 3.1 Anatomical studies

A sharp blade was used to cut the galled leaf segments anatomically, and the thinnest pieces were dyed. The protocol listed below was chosen. Fresh solutions of safranin and methyl blue, each 5% in water, were combined in an equal ratio. The portions of galled segments were submerged in the combination for 10–20 min before being transferred to a solution of 50% alcohol. The sections were moved to 95% alcohol for 5 min, washed with pure alcohol for 5 min, mounted in a drop of glycerol, and dried. Different resolutions of an optical microscope (Lebomed LB-201) were used to observe the sections, and photos were taken using a Vivo S1 Pro camera (Verhertbruggen et al., 2017).

### 3.2 Computational studies

**Figure 1:**
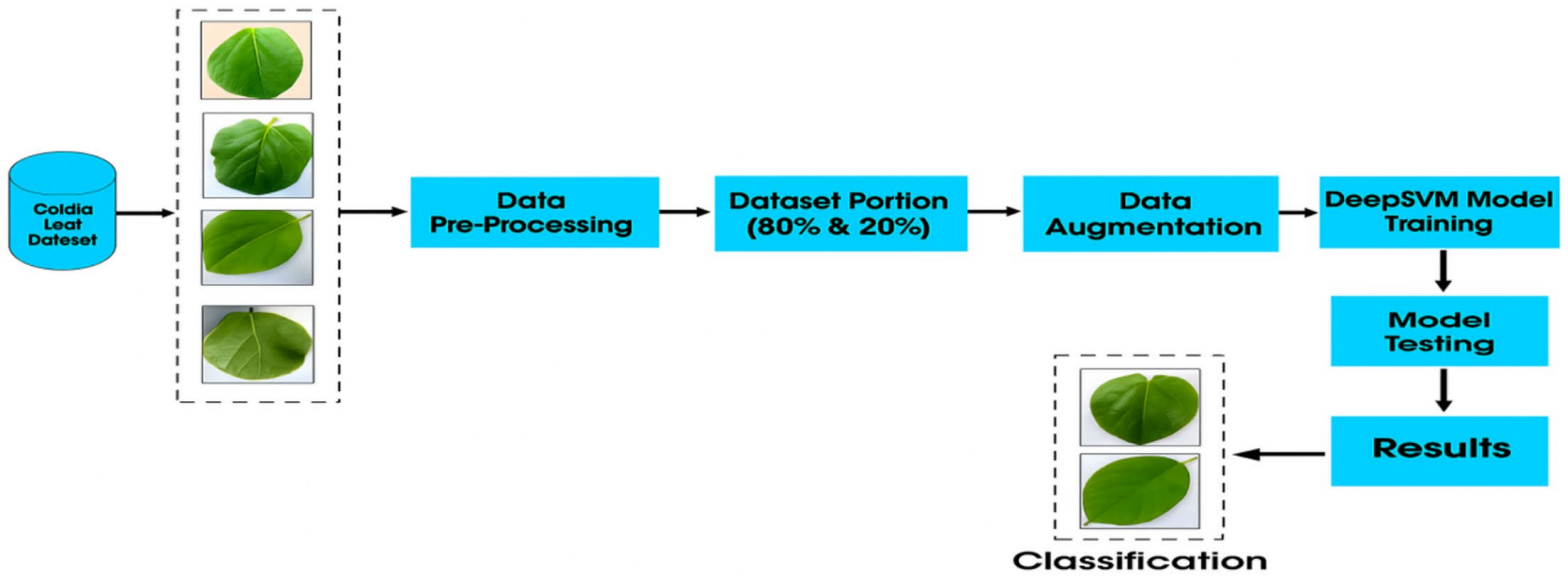
Workflow of the suggested method.

In this study, a novel model named DeepSVM was developed to identify *Cordia dichotoma* leaf images as healthy or diseased. Transfer learning was utilized by using ResNet-50 as the backbone for the model, which allowed us to leverage the pre-trained weights of the ResNet50 model previously trained on a large dataset (ImageNet) for feature extraction. On top of the ResNet50 backbone, three FC layers were added to learn higher-level representations specific to domain image set. Finally, SVM was used for classification as the output layer, a popular choice for classification tasks. The results showed that the proposed approach achieved high accuracy in classifying plant leaf images as healthy or diseased, demonstrating the effectiveness of using transfer learning and SVM in this context. The flow of the model is shown in Figure 1.

### 3.3 Data collection

**Figure 2:**
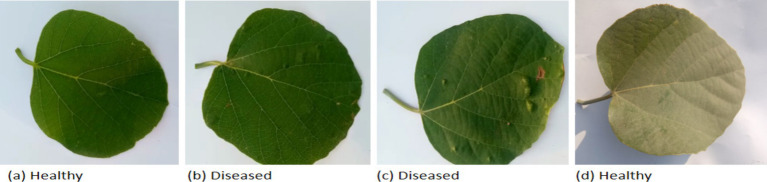
Sample images from training dataset. **(a)** Healthy; **(b)** diseased; **(c)** diseased; **(d)** healthy.

Developing real-time applications using DL necessitates a dataset comprising images for training the model. Unfortunately, there is no online dataset currently available on Cordia leaves to train and validate a DL model aiming to identify healthy and unhealthy Cordia leaves. Therefore, a custom dataset containing 3,500 images was collected carefully for model training, with an additional set of 400 images allocated for testing. A detailed distribution of the dataset is provided in Table 1. The leaf images were manually annotated with binary class labels: Healthy and Dome Galls. The leaves of *Cordia dichotoma* show variations in shape due to several factors, including soil type, overall plant health, climatic conditions, water availability, pest attacks, and nutrient deficiencies. Therefore, the images were collected from diverse locations, including urban and rural areas within the Bannu district of Khyber Pakhtunkhwa (KP), Pakistan, ensured that the dataset accurately reflected real-world scenarios. Most of these images were captured in the semi-hilly Baka Khel subdivision of district Bannu. The original image resolution was 2016 × 4,704 pixels, captured using a TECNO Camon 20 camera (64 MP, f/1.7 wide lens, 2 MP depth sensors). Images were collected under real field conditions at different times of the day (morning, noon, afternoon, and evening) to ensure variability. All images were resized to 256 × 256 pixels for model training. The dataset was split into 80% for training, 20% for validation, and 400 independent test images (200 per class). Importantly, it should be emphasized that employing authentic, real-world images as opposed to pre-existing internet datasets can significantly enhance the performance of DL systems (Nagaraju and Chawla, 2020). A sample of the pictures gathered for the suggested work is shown in Figure 2. The dataset is available on request for the researchers to experiment with the data and further improve the model accuracy.

**Table 1:** Distribution of *Cordia dichotoma* leaf images across training, validation, and test sets.

Image Set	Healthy	Dome galls	Total
Training	1,400	1,400	2,800
Validation	350	350	700
Testing	200	200	400
Total	1950	1950	3,900

### 3.4 Pre-processing

Image preprocessing is crucial step in training a DL classifier for computer vision tasks. Removing inadequate images, including those of poor quality, blurry, or low contrast, from a training dataset is essential in preparing the data for training a DL model. Inadequate images can negatively affect the model’s accuracy and result in poor performance (Li et al., 2025). Each image must be inspected manually to assess its quality and remove inadequate images. It is a time-consuming process, especially if the dataset is large but removing these images can improve the model’s accuracy, ensuring that it’s trained on high-quality data. Image resizing is essential in preparing images for feeding into a DL model. Similarly, CNN-based approaches are sensitive to the shape and size of the input photos. Therefore, all images should be resized to a standard size before being fed into the model. All input images were downsized to 256 by 256 for the proposed study. This resizing is performed using bilinear interpolation, a common method that estimates the pixel value at a non-integer coordinate (x, y) by considering the weighted average of the four nearest neighboring pixels. The interpolated value is computed using Equation (1) as follows:


$$\begin{array}{l} I(x,y)\approx (1-a)(1-b).I\;(x_{0},y_{0}\;)+a(1-b).I(x_{1},y_{0})\\ +(1-a)b.I(x_{0},y_{1})+\mathrm{ab}.I\;(x_{1},y_{1}) \end{array}$$ (1)

Where:

x₀ = ⌊x⌋, x₁ = ⌈x⌉.

y₀ = ⌊y⌋, y₁ = ⌈y⌉.

a = x − x₀, b = y − y₀.

I(x, y): interpolated pixel intensity.

I(x₀, y₀), etc.: intensities of the four neighboring pixels.

Image normalization is a method used in image processing to bring an image’s pixel values into a predetermined range or scale. For many deep learning (DL) algorithms, like Convolutional Neural Networks (CNNs), to learn effectively, the pixel values must fall within a similar range, which is typically achieved through normalization. In the proposed approach, each image was normalized to fall between 0 and 1 by dividing each pixel value by 255, i.e., using the transformation (rescale = 1./255). This converts the original 8-bit pixel values (ranging from 0 to 255) into floating-point values between 0 and 1. These image preparation methods help increase the model’s accuracy and improve its generalization to unseen data.

### 3.5 Data augmentation

**Figure 3:**
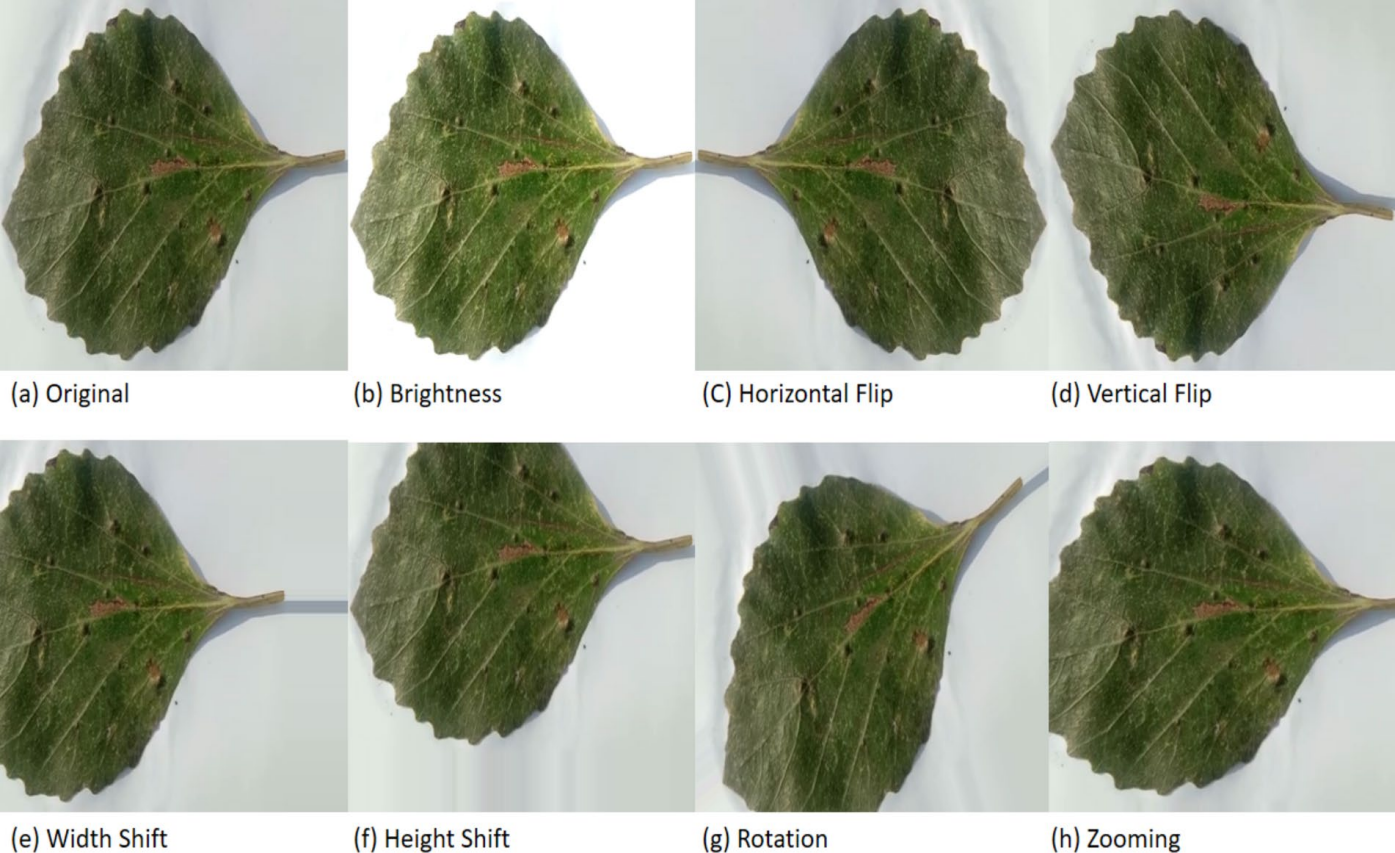
Augmentation methods used in the proposed work. **(a)** Original; **(b)** brightness; **(c)** horizontal flip; **(d)** vertical flip; **(e)** width shift; **(f)** height shift; **(g)** rotation **(h)** zooming.

Data augmentation is a strategy employed to expand the size of a training dataset by applying different modifications to the original images, such as rotation, mirror imaging, cropping, and scaling. Subsequently, the augmented data is employed for training the proposed model. The significance of data augmentation in CNN model training is rooted in its capacity to enhance the model’s ability to generalize. By introducing variations to the source images, the model can learn to identify the same object in diverse configurations and orientations, thereby enhancing its resilience to input variations. Moreover, data augmentation can serve as a countermeasure against over-fitting. Data augmentation reduces the possibility of over-fitting by adding a variety of changes to the data used for training, creating a more relevant and diverse image set. In the proposed approach, transformations like brightness (0.4–1.5), horizontal and vertical flipping, adjustments in height (0.2) and width (0.2), zooming and rotation (30), zooming (0.2) were employed. Some sample images post-augmentation are illustrated in Figure 3.

### 3.6 Proposed DeepSVM approach

**Figure 4:**
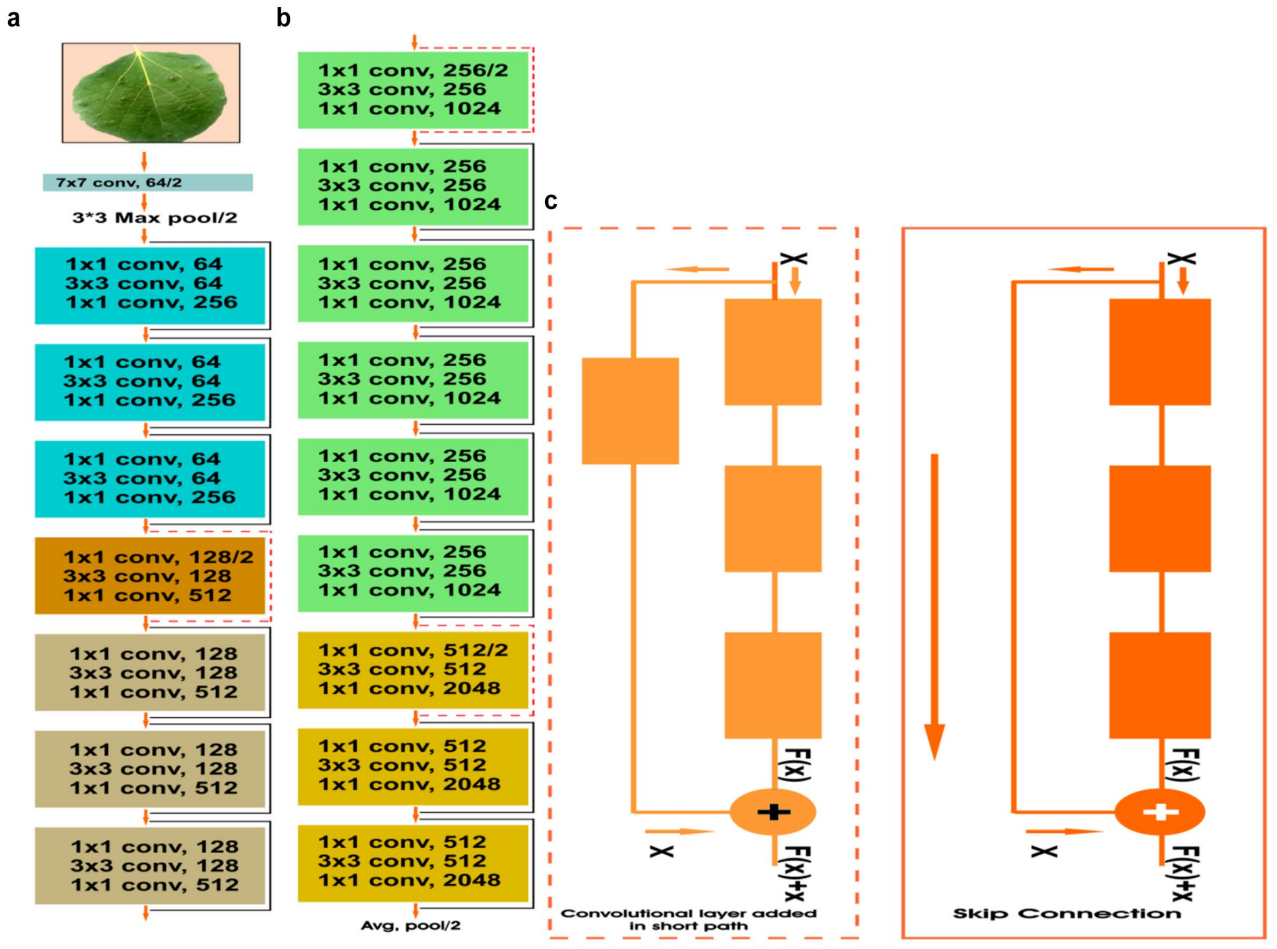
**(a)** General architecture of ResNet-50 **(b)** skip connection (Conv Block) **(c)** skip connection.

The DeepSVM model proposed in this study integrated the Resnet-50 as its pre-trained backbone, augmented with three FC layers and an SVM as the output layer. ResNet-50 is a famous pre-trained model developed by Microsoft researchers in 2015. The general architecture of Resnet-50 is depicted in Figure 4. Its architecture starts with a convolutional layer having 64 feature maps with a 7×7 kernel size, followed by a max pooling layer. It adds sixteen residual blocks with 48 convolutional layers, with three in each block, followed by a global average pooling layer. The number of feature maps in convolutional layers is continuously increasing, going from top to bottom. Convolutional layers operate by applying a filter or kernel to an input matrix or image, performing element-wise multiplication followed by summation, to produce an output matrix known as a feature map. It extracts the features from the input images and then sends them to the FC layers for feature classification which is described as follows:

Let $I={\left[a_{\left(i,j\right)}\right]}_{c\times d}$ be the input matrix of order c × d and $K={\left[k_{\left(m,n\right)}\right]}_{k\times k}$ be the filter of order k (k is odd) is a smaller matrix. The filter slides across the input matrix, and at each position, it performs an element-wise multiplication with the corresponding region of the input. The results are then summed to generate the output matrix, as defined in Equation 2:


$$C_{\left(i,j\right)}=\sum_{m,n=1}^{k}a_{\left(i+m,j+n\right)}*k_{\left(m,n\right)}$$ (2)

Where “m” and “n” denote the positions of the kernel indices, and the sum is computed across these indices.

The ResNet (Residual Network) models are famous for tackling the challenge of vanishing gradients within intricate neural networks by incorporating skip connections. There are two types of skip connections, identity blocks or identity skip connections and convolutional blocks or projection skip connections. Identity skip connections are the basic skip connections where the input to a layer is added directly to its output as shown in Figure 4c. In ResNet-50, these connections are used in the residual blocks where the input and output dimensions are the same. Projection skip connections are utilized when the dimensions of the output and input of a residual component are not equal. In such cases, a 1×1 convolutional layer is employed to adjust the dimensionality of the input so that it matches the output dimension shown in Figure 4b. These connections are used in certain residual blocks within ResNet-50 where down sampling is required. The skip connections, alternatively labeled as shortcut connections, serve as a mechanism within deep neural networks to mitigate the vanishing gradient issue. In a neural network, gradients are employed for weight updates during training. However, as the gradient is propagated through the network, it can become very small, especially in deep networks, making it difficult to update the weights effectively. This can lead to slower convergence or the network becoming stuck in a local minimum. Skip connections aim to solve this issue by providing an alternate, more direct way for the gradient to pass via the network. This is achieved by adding a connection that skips one or more layers in the network. It works as follows:


Y=F(x)+x (3)

Equation 3 shows how a neural network layer works: x is the input, and Y is the output. The function F(x) processes the input to extract useful information, and then the original input x is added back to the result of F(x), piece by piece. This helps the network learn more effectively by keeping some of the original information.

ResNet-50, having undergone extensive training on the ImageNet dataset with over 1.2 million images across 1,000 classes, brings a wealth of diverse features to the model. This pre-training on ImageNet establishes ResNet-50 as a popular choice for transfer learning in various computer vision tasks such as object detection, image segmentation, and classification. Transfer learning exploits the knowledge gained during pre-training, allowing fine-tuning on a new dataset or task with limited labeled examples. This approach often leads to enhanced performance and faster convergence, especially when dealing with smaller datasets. The transfer learning process involves using the pre-trained model’s weights as a starting point, replacing the final layer(s) with task-specific ones, and then training these new layers on the new dataset. In contrast, the pre-trained layers are frozen or fine-tuned with a reduced learning rate. In the proposed study, three FC layers were used for feature classification. Each FC layer was followed by a drop-out layer to reduce over fitting. SVM was used as the output layer for disease identification with L2 regularization.

SVM is the final/output layer for classifying the disease called dome galls. SVM, an ML technique, is employed for classification and regression analysis tasks. In classification, SVM endeavors to segregate data instances into distinct categories by identifying the hyper plane that optimizes the separation margin between these categories. This hyper plane serves as the boundary, ensuring the greatest possible gap between data instances of disparate classes. SVM is a potent algorithm that addresses linear and nonlinear classification and regression quandaries. Its widespread adoption spans diverse domains, including image classification, textual categorization, and bioinformatics. In the proposed approach, SVM is employed for binary classification using the hinge loss function along with L2 regularization. The mathematical representation is provided in Equation 4 below:


$$\mathrm{minimize}:\frac{1}{2}{||\omega ||}^{2}+C\sum (\max(0,1-y_{i}(\omega^{T_{x_{i}}}+b)))$$ (4)

In this context, *ω* represents the weight vector, b is the bias term, and C is the regularization parameter that controls the trade-off between maximizing the margin and minimizing classification errors. yᵢ denotes the binary class label for the *ith* training example, while xᵢ is its corresponding feature vector. The symbol ∑ indicates summation over all training examples. With these definitions, the hinge loss function used in the L2-regularized binary SVM is expressed in Equation 5 as follows:


$$\max(0,1-y_{i}(\omega^{T_{x_{i}}}+b))$$ (5)

This loss function imposes a penalty on the model when a training sample is misclassified or falls on the wrong side of the decision boundary. If the model correctly classifies a sample with sufficient confidence, the loss becomes zero. The hinge loss is a convex function, meaning it has a single global minimum, and while it is not differentiable exactly at the point where the argument of the max function is zero, it remains smooth and manageable for optimization elsewhere. Table 2 contains the architecture and training configuration of the proposed DeepSVM Framework.

**Table 2:** Model architecture and training configuration of the proposed DeepSVM framework.

S. No	Layer	Neurons/Value	Parameter	Value
1	Backbone (ResNet-50)	Transfer Learning	Input Image Size	256 × 256
2	Flatter Layer		Activation Function	ReLU
3	FC layer	512	Optimizer	SGD
4	Dropout	0.3	Momentum Value	0.9
5	FC Layer	256	Number of Classes	2 (Healthy, Dome Galls)
6	Dropout	0.3	Batch Size	32
7	FC Layer	128	Training Epochs	98
8	Dropout	0.3	Loss Function	Hinge
9	Final Layer (SVM)	1	Regularization	L2, (Used in final layer only)
10			Learning Rate	0.0001
11			Early Stopping	Yes

## 4 Results and discussion

### 4.1 Training environment

In this study, Google Colab was used to develop and train the models. Google Colab offers a cost-effective alternative, providing a free GPU-enabled environment. Integrated with Google services like Gmail and Drive, it allows easy access to datasets and models. Its interactive notebooks, equipped with pre- installed libraries, streamline coding, saving time on setup. The Tesla T4 GPU, with 12 GB of RAM, powers Colab, accelerating model training for faster convergence and improved results. Keras library was used for developing the CNN models. This combination democratizes advanced hardware access, empowering researchers in image analysis with an efficient, collaborative platform.

### 4.2 Performance measurement metrics

To assess the model’s effectiveness, we employed a quartet of distinct metrics: accuracy, precision, recall, and the F1-score (Srinivasu et al., 2025). Accuracy is a conventional measure, gauging the global accuracy of the model’s predictions, shown in Equation 6. On the other hand, precision and recall gauge the model’s aptitude in accurately distinguishing positive and negative samples, shown in Equations 7, 8, respectively. The F1-score, a harmonic amalgamation of precision and recall, furnishes a harmonious gauge of the model’s performance, shown in Equation 9. Specificity, which measures the proportion of correctly identified negative samples, is shown in Equation 10.


$$\text{Accuracy}=\frac{\mathrm{TN}+\mathrm{TP}}{\text{Total Samples}}$$ (6)

$$\text{Precision}=\frac{\mathrm{TP}}{\mathrm{TP}+\mathrm{FP}}$$ (7)

$$\text{Sensitivity}\;(\text{recall})=\frac{\mathrm{TP}}{\mathrm{TP}+\mathrm{FN}}$$ (8)

$$F_{1}-\text{Score}=2(\frac{\text{Precision}\times \text{Recall}}{\text{Precision}+\text{Recall}})$$ (9)

$$\;\text{Specificity}=\frac{\mathrm{TN}}{\mathrm{TN}+\mathrm{FP}}$$ (10)

Where true positives (TP) show the correct classification of dome galls; true negatives (TN) show the correct classification of healthy leaves; false positives (FP) show the wrong classification of dome galls, i.e., healthy leaves as dome galls; and false negatives (FN) show the wrong classification of healthy leaves as dome galls.

### 4.3 Experiments and results

#### 4.3.1 Anatomical results for dome galls

**Figure 5:**
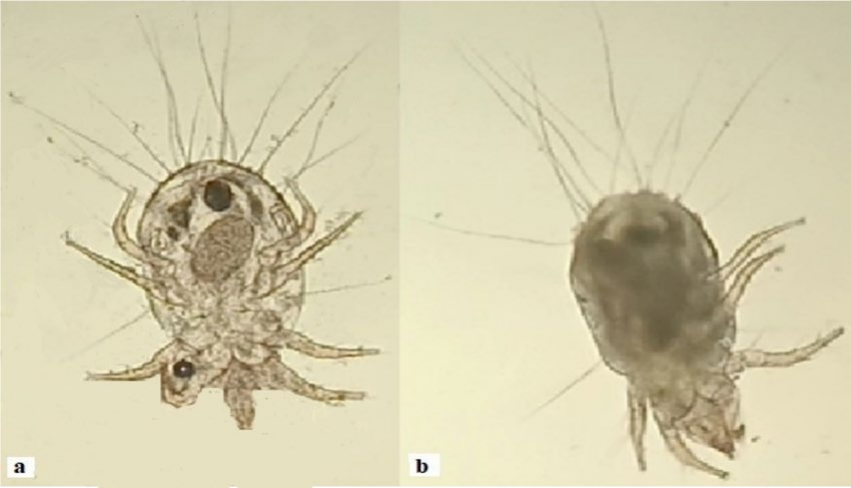
**(a)** Mite, surface morphology **(b)** side view.

**Figure 6:**
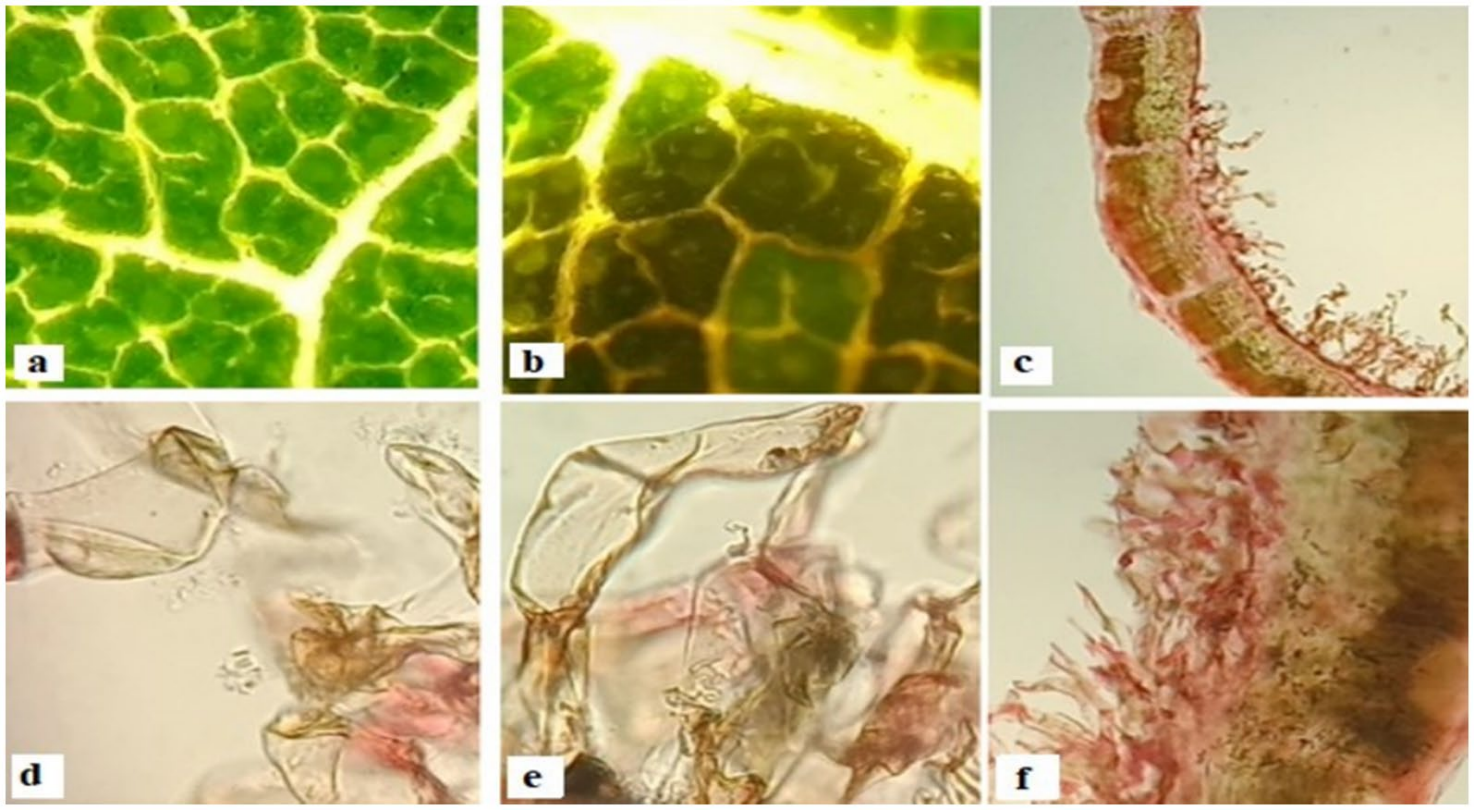
**(a)** Healthy leaf section **(b)** initiation of dome gall **(c)** transverse section of leaf gall segment **(d)** trichome **(e)** trichome, closer view **(f)** Multiple trichomes.

**Figure 7:**
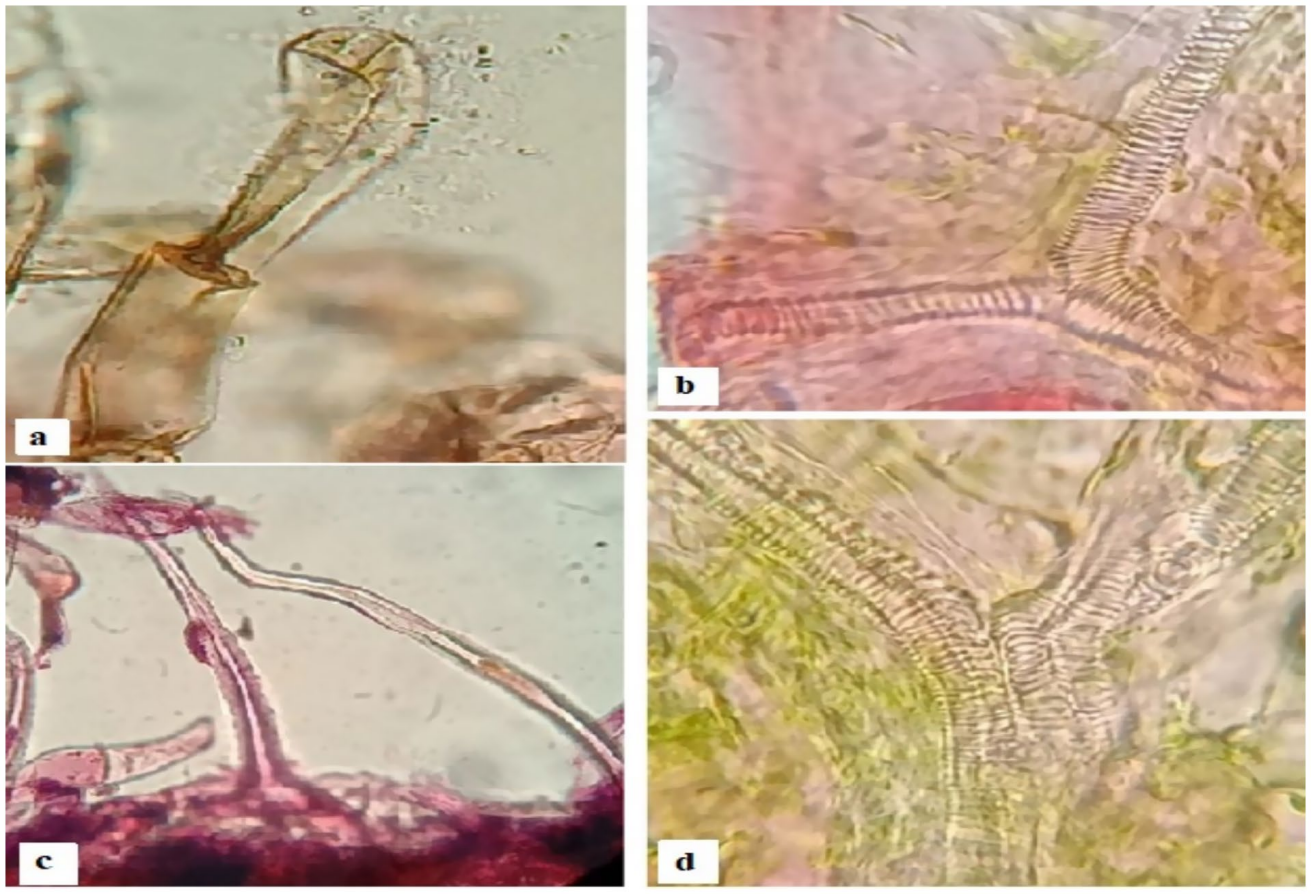
**(a)** A trichome with its secretion **(b)** irregular xylem vessel **(C)** multiple trichomes **(d)** multiple.

A tiny mite (Tyrophagus putrescentiae) was isolated from galled tissues in *Cordia dichotoma*, as shown in Figure 5. The mite may be the possible inducer of dome galls in *C. dichotoma*. A diversity of calcium oxalate crystals in mites-induced gall tissues in Cordia was observed Figure 6. These crystals are proposed to be released by the plant tissues as a defense against mites-induced stress. Anatomical observations of transverse sections of the leaves indicated the development of various trichomes. The trichomes are proposed to be developed by the plant as physical barriers to the mites. Irregular extensions of vascular tissues were noticeable (Figure 7).

**Figure 8:**
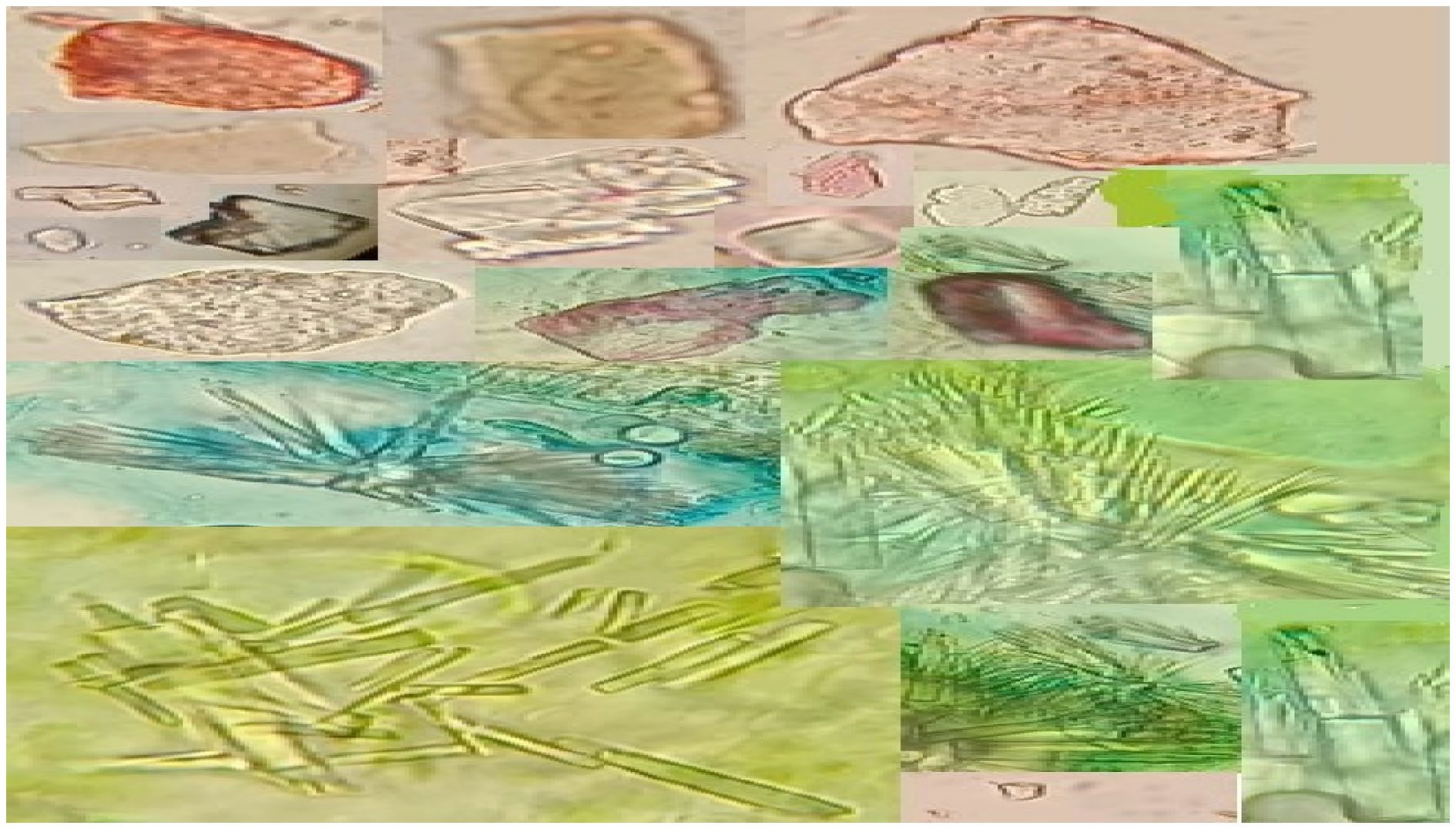
Calcium oxalate crystals of various dimensions.

In plants, Galls are anomalous fleshy or woody outgrowths that are sometimes referred to as warts and tubercles when they are small or knots when they are large, usually with a web of complex and branched vascular tissues distributed irregularly (Lu et al., 2019). During tumor induction, cell hypertrophy is usually the initial noticeable response of the host plant organ (dos Santos Isaias et al., 2014). The presence of calcium oxalate crystals of varying shapes and dimensions within gall tissues is illustrated in Figure 8.

The gall tissues are often accompanied by trichomes and other modified cells, which act as physical barriers to possibly, protect the tissues from further damage (Ferreira and dos Santos Isaias, 2014). Plant galls are usually distinct structures often induced by the invasion of pathogenic organisms, like insects, mites, nematodes and microbes on plants (Anand and Ramani, 2021). The initiation of a gall is accompanied by the rapid cell division and differentiation of parenchyma cells in order to provide supplementary vasculature to the growing gall. Dolzblasz et al. (2018) reported that leafy galls develop a complex network of vascular tissues in order to ensure the transport of water and dissolved minerals to the growing apices of gall. Karabourniotis et al. (2020) have reported anatomical and chemical modification in trichomes and structural diversity in trichomes as a plant’s strategy to overcome biotic and abiotic stresses. Moreover, they also reported that trichomes function as physical barriers to protect plant tissues against foreign invaders. While Nakata (2012) reports that plant synthesizes a diversity of calcium oxalate crystals when they are under stress. These crystals have been reported to play a vital role in regulating cellular calcium and protecting plants from biotic and abiotic stresses (Gómez-Espinoza et al., 2021).

#### 4.3.2 Results with the proposed DeepSVM framework

In this study, a novel model, DeepSVM, was trained and fine-tuned, leveraging a dataset of 3,500 images for the accurate identification of early symptoms related to dome galls, a prevalent leaf disease in Cordia plants. The early symptoms of dome galls are similar to those of healthy leaves and require more concentration to classify. The model’s architecture culminated in a powerful configuration featuring Resnet-50 as its backbone, three FC layers, and an SVM as the final output layer. Training extended to 98 epochs, implementing the effective technique of early stopping to mitigate over-fitting risks by discontinuing training upon validation set performance degradation. Rigorously evaluated on 400 previously unseen images, the model showcased high performance with an accuracy of 94.50%. Comprehensive results are presented in Table 3. The results demonstrate that the model achieved high accuracy, precision, recall, and F1-score, indicating its effectiveness in classifying plant leaves as healthy or diseased. These findings suggest that the developed model could be an invaluable tool for diagnosing plant disease, helping farmers and agricultural experts identify and treat diseased plants promptly.

**Table 3:** Shows the outcomes of DeepSVM on the test images.

Serial No	Metric	Value (%)	SD%
1	Accuracy	94.50	1.50
2	Precision	96.00	–
3	Recall	93.00	–
4	Specificity	96.00	–
5	F1-Score	94.47	1.40

In this experiment, a comprehensive exploration of model training strategies for the early symptom identification of dome galls was undertaken. Employing deep and shallow architectures, experiments ranged from training models from scratch to utilizing transfer learning. The customized CNN model, featuring seven convolutional layers with BN after each block, two FC layers, dropout layers, and an output layer, served as the baseline for the proposed research, with detailed results in Table 4.

**Table 4:** Performance comparison of DeepSVM with other models.

Model	Accuracy (%)	Precision (%)	Recall (%)	F1-Score (%)	Parameters (millions)	GFLOPs
MobileNetV2	91.33	93.76	88.23	90.91	3.40	0.30
VGG-16	90.87	92.37	87.00	89.60	14.7	15.0
VGG-19	91.80	92.44	90.74	91.58	20.0	19.0
InceptionResNet-v2	92.65	94.77	92.88	93.79	55.9	12.5
Custom-CNN	87.41	87.74	83.37	85.49	3.34	2.00
DeepSVM	94.50	96.00	93.00	94.47	25.6	3.80

**Figure 9:**
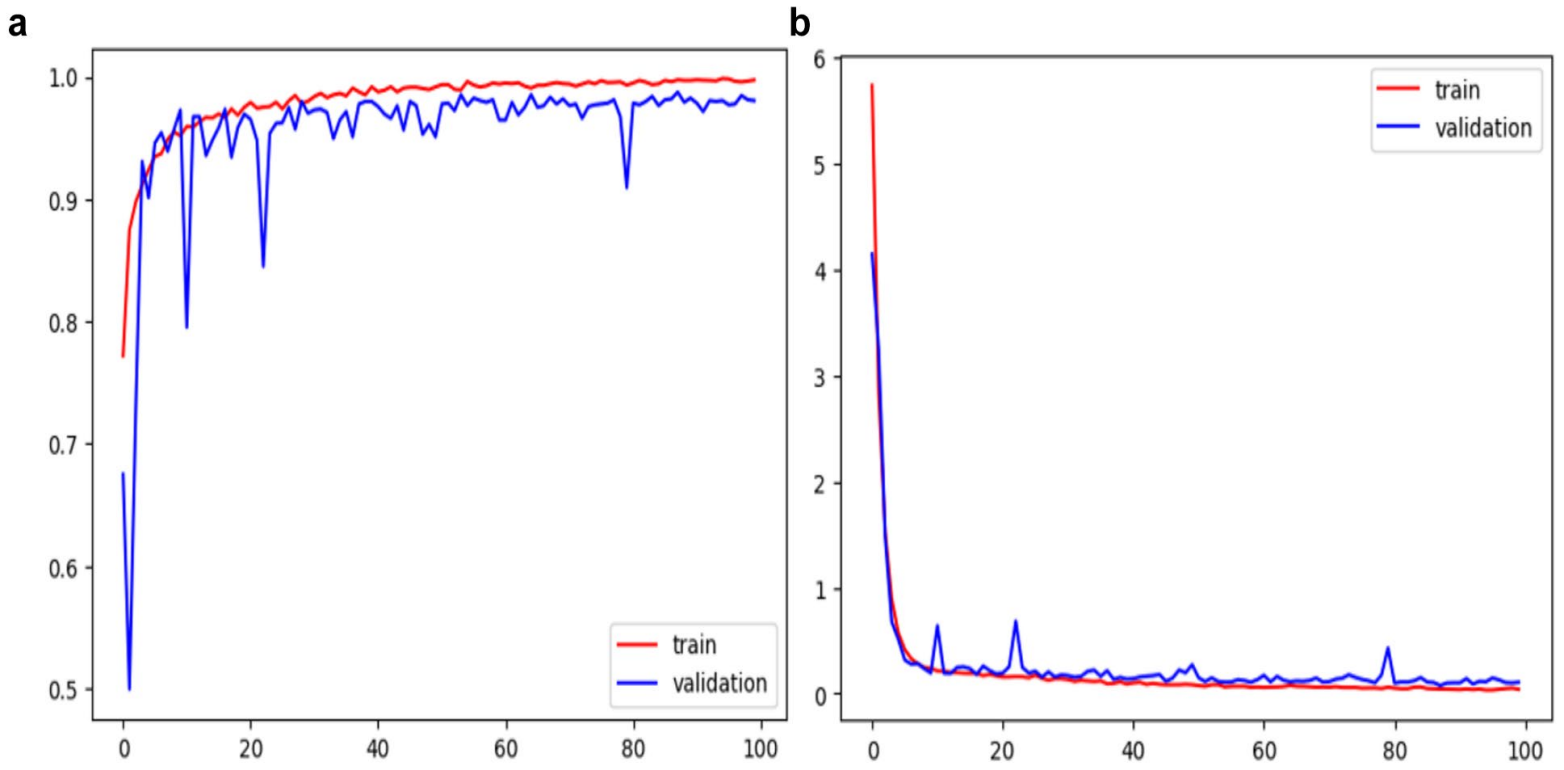
Shows training performance of the proposed DeepSVM **(a)** accuracy **(b)** loss.

**Figure 10:**
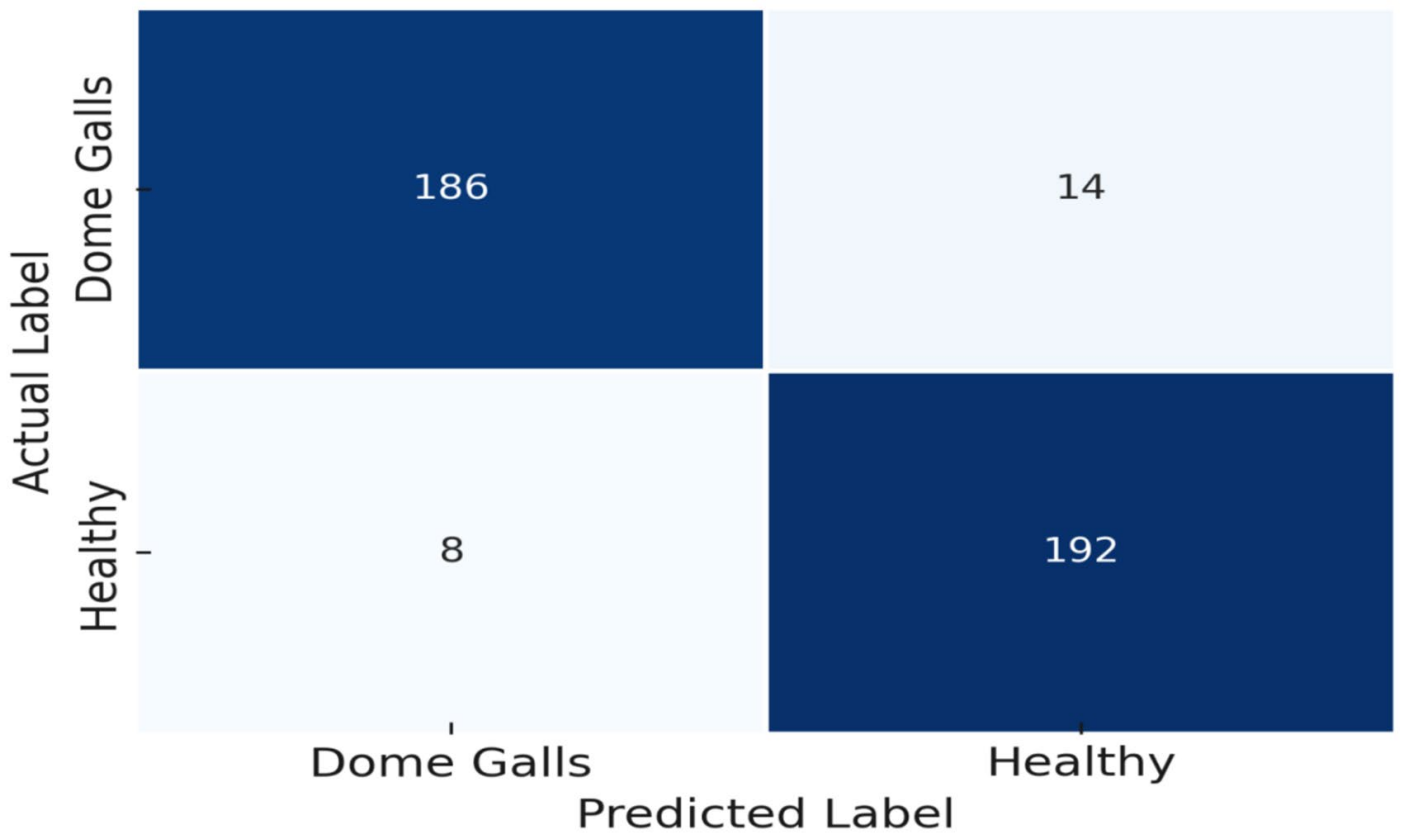
Shows confusion matrix of the proposed model.

The concept of training oscillation in DL, denoting erratic fluctuations in metrics like loss or accuracy during training, was elucidated. This phenomenon often arises from issues such as inappropriate learning rates or model architecture, impeding the convergence of the training procedure. Incorporating L2 regularization in the final layer effectively aligned results with the research objectives. Visual representations of the training and validation performance, encompassing accuracy and loss functions, are depicted in Figure 9. Additionally, MobileNet-v2 (Sandler et al., 2018), VGG-16 (Simonyan and Zisserman, 2014), InceptionResNet-V2 (Naveenkumar et al., 2021) and VGG-19 (Simonyan and Zisserman, 2014) were trained with the same parameters and FC layers, and their respective results are outlined in Table 4. Instead of three FC layers, the custom CNN model showed good performance with two FC layers. The confusion matrix of the proposed model showing detailed of TPs, TNs, FPs and FN is depicted in Figure 10.

The DeepSVM, employing ResNet-50 as a backbone, demonstrated strong performance in classifying *Cordia dichotoma* leaf images as healthy or infected. The utilization of ReLU activation, SGD optimizer with momentum (0.9), and hinge loss function contributed to the model’s success. ResNet-50’s deep architecture with 50 layers allowed it to learn complex features, addressing the vanishing gradient issue through residual connections. These connections facilitated efficient gradient propagation and reduced the risk of over-fitting. The model incorporated three FC layers, progressively reducing neurons (512, 256, and 128) toward the output layer, with dropout layers (dropout ratio of 0.3) and L2 regularization in each FC layer. This design enabled the model to capture intricate nonlinear relationships between features and class labels. The final layer employed SVM, outperforming sigmoid, showcasing SVM’s effectiveness in handling complex data and generalizing well to unseen instances. The DeepSVM with ResNet-50 as a backbone and SVM as the final layer presented a robust solution for accurate leaf image classification tasks (Tang, 2013).

### 4.4 Ablation study

The proposed model, DeepSVM, underwent a series of iterative experiments involving continuous adjustments to its architecture and hyper parameters. In the proposed study, transfer learning was used, and the main focus was on the head (FC layers) of the CNN model, changing the number of FC layers followed by the number of neurons per layer. The model was initially trained with a single fully connected (FC) layer containing 1,024 neurons, followed by experiments with two FC layers comprising 512 and 256 neurons, respectively. The best results, however, were achieved using three FC layers with 512, 256, and 128 neurons, respectively. Most of the previous literature used various optimizers, including Adam and stochastic gradient descent (SGD). In this study both of them were used, but SGD with momentum (0.9) performed well compared to Adam. The learning rate was adjusted multiple times during experimentation, with the optimal performance achieved at a finalized value of 0.0001. To reduce over fitting, the L2 regularizer was used in the final layer with a sigmoid activation function. After incorporating three fully connected (FC) layers with SGDM (momentum = 0.9), a loss value of 0.0001, a sigmoid activation function, and an L2 regularizer, a notable improvement was observed in training and validation accuracy as well as loss values. However, a non-negligible amount of oscillation persisted in the training and validation curves, potentially affecting the model’s generalization in real-world applications. To overcome this issue, the sigmoid activation function was replaced by SVM which greatly enhanced the results with reduced oscillation. Therefore, a model that contained a backbone of 50 convolutional layers, three FC layers and an SVM as an output layer was named DeepSVM.

### 4.5 Performance evaluation of the DeepSVM on public datasets

The results of the suggested model on two publicly accessible datasets show that it is somewhat more adaptable and performs better in a couple of areas. The model demonstrated an improved precision in classifying plant leaf illnesses using the Potato Leaf Disease (PLD) dataset, which consists of 4,072 images spanning the healthy, early blight, and late blight classes. On the pulmonary X-ray image dataset, which comprises 6,432 medical images classified into three classes—normal, COVID-19, and pneumonia—the model’s stated accuracy of 98.00% was similarly intriguing. This improved performance revealed the model’s capacity to recognize intricate patterns within radiography images, creating intriguing opportunities to enhance medical diagnostic processes. By carefully assessing its performance metrics, as indicated in Table 5, the model’s versatility and efficacy in addressing the unique issues presented by detecting plant diseases and analyzing medical images were brought to light. These findings demonstrated the improved generalizability of the suggested approach and not only demonstrated its adaptability but also it’s potential to make a substantial contribution to healthcare and agriculture.

**Table 5:** Performance assessment of the DeepSVM on public datasets.

Dataset	Source	Accuracy (%)	Precision (%)	Recall (%)	F1-Score (%)
PLD	Kaggle	97.50	98.50	96.53	97.42
Chest X-Ray	Kaggle	98.00	97.25	97.50	97.37

### 4.6 Comparison of the DeepSVM with other SOTA methods

The proposed approach is evaluated on a novel dataset curated for the dome galls. We cannot find related studies to evaluate and compare the performance of the proposed approach with the SOTA approaches concentrating on similar datasets. Therefore, to identify the effectiveness and performance of the proposed DeepSVM approach, it is evaluated against the publicly available dataset. The DeepSVM was trained and tested on the PLD dataset to create a solid foundation for the work. The detailed results are shown in Table 6. The model exhibited better performance, achieving a test accuracy of 97.50%. This achievement served as a testament to the efficacy of the novel approach. A meticulous comparative analysis was conducted to provide a comprehensive perspective, pitting the model against prior research that employed the same publicly available dataset. The detailed outcomes of this comparative evaluation are meticulously outlined in Table 4, offering valuable insights into the significant advancements made by the suggested model (Table 6).

**Table 6:** Comparison of DeepSVM method with previous work on a public dataset.

Study	Method	Dataset	Year	Accuracy (%)
Islam et al. (2017)	CNN	Potato Leaves	2017	92.00
Rozaqi and Sunyoto (2020)	ANN	Potato Leaves	2020	96.50
Sanjeev et al. (2021)	Segmentation + MSVM	Potato Leaves	2021	95.00
Singh and Kaur (2021)	K-Means + MSVM	Potato Leaves	2021	95.99
Kurmi et al. (2022)	CNN	Potato Leaves	2022	95.35
Mahum et al. (2023)	Efficient-Net DenseNet	Potato Leaves	2023	97.20
Ashikuzzaman et al. (2024)	DenseNet201	Potato Leaves	2024	96.00
DeepSVM	CNN + SVM	Potato Leaves	–	97.50

### 4.7 Limitations of the study

In the context of our proposed study, DeepSVM, a novel model meticulously crafted for the early symptom identification of dome galls, showcased better results by achieving a better accuracy of 94.50% on the test dataset. This accomplishment represented a significant stride forward in the domain of plant leaf disease classification. However, an avenue for improvement lies in enhancing computational efficiency. DeepSVM necessitated a longer training duration than contemporaneously trained models, particularly those employing the sigmoid activation function as the final layer and Adam optimizer. The forthcoming research endeavors will optimize the DeepSVM training process and explore alternative optimization strategies, including optimizers and other parameters. This pursuit aims to strike an optimal equilibrium between elevated accuracy and diminished computational time, thereby fortifying the model’s practical applicability in real-world scenarios. Moreover, the proposed DeepSVM approach is a black box, which takes dome galls images as inputs and predicts whether the input image is healthy or unhealthy. To better understand the results of the proposed DeepSVM approach, we aim to introduce eXplainble Artificial Intelligence (XAI) with the proposed approach to reduce its complexity and results fairness.

## 5 Conclusion and future work

This study aimed to create a model that can spot early signs of dome galls, a leaf ailment that affects Cordia plants, and introduced a new method called DeepSVM. The model was built using ResNet-50 as the backbone, three FC layers, and the final layer contained SVM instead of the sigmoid activation function. A custom dataset containing 3,500 images of healthy and diseased leaves with class balancing was used for training the model, equipped with preprocessing and data augmentation techniques to enhance generalization and reduce over fitting, resulting in a test accuracy of 94.50%. The study highlighted the model’s potential for improving plant disease detection by highlighting its effectiveness in early symptom classification for plant leaf diseases. The proposed approach offers a reliable tool for early plant disease detection, enhancing crop productivity and outperforming traditional algorithms. It encourages the integration of anomalous histological feature extraction with the DeepSVM model to enhance performance further. This framework can be extended to other agricultural applications, improving existing machine and deep learning methods. Ultimately, it supports farmers in selecting effective pesticides, reducing costs, and maintaining crop quality through timely diagnosis.

There is scope for further research to improve the performance of the proposed approach. The accuracy may be increased, and the training time may be reduced without compromising accuracy. Collaborating with domain experts to amass a more comprehensive and diverse dataset, stratified into subcategories such as initial, mature, and severe dome gall cases, would enhance method evaluation. Expanding the proposed method to encompass various plant species for early leaf disease symptom identification holds promise. Moreover, exploring an extension of this approach to classify human diseases could broaden its applicability, fostering advancements in plant and human disease classification techniques.

## Data Availability

The raw data supporting the conclusions of this article will be made available by the authors, without undue reservation.
